# Analysis of the Clinical Efficacy of Laparoscopy and Hysteroscopy in the Treatment of Tubal-Factor Infertility

**DOI:** 10.3389/fmed.2021.712222

**Published:** 2021-08-16

**Authors:** Lei Nian, De-Hong Yang, Jie Zhang, Han Zhao, Cai-Fen Zhu, Ming-Feng Dong, Ying Ai

**Affiliations:** ^1^Department of Obstetrics and Gynecology, The First People's Hospital of Yunnan Province, Kunming, China; ^2^The Affiliated Hospital of Kunming University of Science and Technology, Kunming, China

**Keywords:** laparoscopy, hysteroscopy, tubal infertility, pregnancy rate, clinical efficacy

## Abstract

**Objective:** This study aims to investigate the clinical efficacy of laparoscopy and hysteroscopy in the treatment of tubal-factor infertility (TFI) to provide a basis for predicting postoperative pregnancy rates.

**Methods:** The clinical data of 336 patients who underwent laparoscopy and hysteroscopy for TFI between February 2018 and December 2018 in the Department of Reproductive Gynecology at the First People's Hospital of Yunnan were retrospectively analyzed. After implementing the inclusion and exclusion criteria, 278 patients were included in the study. The patients were grouped according to pelvic adhesions, hydrosalpinx, twisted fallopian tubes, and fimbriae structure. The impact of the extent of fallopian tube diseases on postoperative pregnancy outcomes was analyzed.

**Results:** Of the 278 patients, 129 got pregnant (pregnancy rate = 46.4%). Pelvic adhesions, hydrosalpinx, twisted/folded fallopian tubes, and damage to the fimbriae of the fallopian tubes were found to affect the natural pregnancy rate after surgery, and it decreased significantly with the aggravation of the disease (*P* < 0.001). Of the 129 patients who had natural pregnancies, 29 had ectopic pregnancies (ectopic pregnancy rate = 22.48%). Twisted/folded fallopian tubes and damage to the fimbriae structure significantly increased the incidence of postoperative ectopic pregnancy (*P* < 0.001).

**Conclusion:** Laparoscopy and hysteroscopy are effective treatments for TFI. Pelvic adhesions, twisted/folded fallopian tubes, hydrosalpinx, and damage to the fimbriae of the fallopian tubes can affect postoperative pregnancy outcomes and lead to failure of a natural pregnancy after the operation. The incidence of ectopic pregnancy increases with the degree of fallopian tube twisting/folding and the degree of damage to the fimbriae of the fallopian tubes.

## Introduction

Tubal-factor infertility (TFI) is induced by changes in the structure and function of the fallopian tubes. These changes are mainly caused by chronic salpingitis, peritubal inflammation, and abnormal fallopian tube development, which reduce peristaltic function and affect egg picking, fertilization, and the transport of fertilized eggs (1). Since Fervers first performed laparoscopic adhesion lysis in 1933 and applied endoscopic technology to the treatment field, laparoscopy and hysteroscopy have become the most commonly used treatments for TFI worldwide.

Many Chinese and foreign scholars have conducted detailed studies on the application of laparoscopy to explore the causes of infertility and the curative effects of treating female infertility (2). Laparoscopic treatment of TFI has several advantages, including less trauma, less bleeding, faster recovery, and a shorter hospital stay (3, 4). Many scholars believe that the laparoscopic diagnosis and treatment of pelvic inflammatory disease, pelvic adhesions, endometriosis, and other pelvic disease-associated infertility is safe and effective (5, 6). However, the postoperative follow-up shows that some patients still experience unsatisfactory pregnancy outcomes. Therefore, the present study followed up on the pregnancy outcomes of patients with TFI to explore the clinical value of laparoscopy and hysteroscopy for the treatment of TFI and provide a basis for predicting the postoperative pregnancy outcomes of TFI and formulating further treatment plans.

## Methods and Data

### General Information

The clinical data of 336 patients who underwent laparoscopy and hysteroscopy for TFI between February 2018 and December 2018 in the Department of Reproductive Gynecology at the First People's Hospital of Yunnan were retrospectively analyzed. After case selection according to the inclusion and exclusion criteria, 278 patients were included in the analysis. All patients underwent combined hysteroscopy and laparoscopy, and none shifted to laparotomy during the treatment. The ages of the patients ranged from 20 to 40 years. The period of infertility was 1–14 years, with an average duration of 4 years. Infertility patterns included 128 cases (46.0%) of primary infertility and 150 cases (54.0%) of secondary infertility. There were no significant statistical differences between the groups in the above-mentioned indicators.

### Inclusion and Exclusion Criteria

#### Inclusion Criteria

(1) Patients met the diagnostic criteria for infertility (1) (partners had lived together and had a normal sex life for a year without contraception and had not been pregnant); (2) aged between 20 and 40 years old; (3) voluntarily chose laparoscopy and hysteroscopy; (4) signed a consent form for surgery; (5) no contraindications for surgery; (6) normal menstruation; (7) intraoperative laparoscopy confirmed the presence of fallopian tube disease.

#### Exclusion Criteria

(1) The male partner had been diagnosed with male infertility; (2) presence of immune factors; (3) abnormal ovulation was found through examinations like sex hormone testing and monitoring of ovulation or luteal function; (4) presence of uterine factors, including genital tract malformations, submucosal fibroids, endometritis, endometrial polyps, endometrial tuberculosis, and intrauterine adhesions; (5) presence of cervical factors, such as cervical inflammation, abnormal cervical mucus secretion, and cervical fibroids; (6) pelvic endometriosis, tuberculosis, or acute inflammation; (7) endocrine diseases, including hyperthyroidism, hypothyroidism, polycystic ovary syndrome, hyperprolactinemia, amenorrhea, and premature ovarian failure.

### Grouping Criteria

#### Degree of Pelvic Adhesion

Adhesion degree criteria were established according to the modified American Society for Reproductive Medicine (ASRM) adhesion grading standards (7). Evaluation criteria and classification: no adhesion (score: 0); mild adhesion: membranous, <25% (score: 1); membranous, 25–50% (score: 2); moderate adhesion: membranous, ≥5% (score: 3); dense, <25% (score: 4); severe adhesion: dense, 25–50% (score: 5); dense, ≥5% (score: 6).

#### Degree of Hydrosalpinx

When the end-pipe diameter of the hydrosalpinx was <1.5 cm, it was defined as mild hydrosalpinx; when it was 1.5–3.0 cm, it was defined as moderate hydrosalpinx, and when it was >3.0 cm, it was defined as severe hydrosalpinx.

#### Degree of Fallopian Tube Twist

The angle of fallopian tube distortion in each patient was visually observed under a laparoscope and the minimum angle recorded. When the minimum angle was ≤45°, it was defined as fully folding, when it was 45–120°, it was defined as partially folding, and when it was ≥120°, it was defined as no fallopian tube distortion.

#### Fimbriae structure of the fallopian tubes

The structure of the fimbriae was categorized according to whether the fimbriae mucosa tissue of the fallopian tubes was damaged or not, which was identified during laparoscopy. When the fimbriae mucosa were intact and the color was normal, the patients were defined as having intact fimbriae. Those patients in whom only part of the fimbriae mucosa tissue was present and in whom the fimbriae tissue was partially wrapped and adhered were defined as having retained fimbriae. Those without fimbriae mucosa tissue or very little fimbriae mucosa tissue were defined as having fimbriae structure destruction.

### Surgical Procedures

All patients underwent hysteroscopy, tubal intubation, and laparoscopic exploration. Those in whom pelvic adhesions were identified during laparoscopy underwent pelvic adhesion lysis surgery, those with twisted/wrapped fallopian tubes underwent plastic surgery and repair of the fallopian tube, those with fimbriae atresia and hydrops underwent salpingostomy, and those with proximal fallopian tube obstruction underwent hysteroscopic tubal intubation and guide-wire intervention.

### Statistical Analysis

The data were imported into SPSS 17.0 software for analysis. Percentages were compared using a χ^2^ test. The inspection level was α = 0.05. *P* < 0.05 was considered statistically significant.

## Results

### Results of Hysteroscopy and Laparoscopy

Of the 278 patients included in the study, 206 had pelvic adhesions (74.1%), 159 had twisted/wrapped fallopian tubes (57.1%), 99 had hydrosalpinx (35.2%), and 190 had abnormal fimbriae structure, including fallopian tube atresia, abnormal development of the fimbriae, and distal obstruction (68.3%).

### Pelvic Adhesion and Postoperative Pregnancy Outcomes

Of the 206 patients with pelvic adhesions, 86 had mild adhesion, 62 of which had a spontaneous pregnancy within 2 years after the operation (natural pregnancy rate = 72.09%). Another 77 patients had moderate adhesion, 40 of which had a spontaneous pregnancy within 2 years after the operation (natural pregnancy rate = 51.95%), and a total of 43 patients had severe adhesion, 12 of which had a spontaneous pregnancy within 2 years after the operation (natural pregnancy rate = 27.91%). These results show that the natural pregnancy rate decreases as the degree of pelvic adhesion increases. The difference was statistically significant (χ^2^ = 23.218, *P* < 0.001) (see Table 1).

**Table 1:** Pelvic adhesion and postoperative pregnancy outcome.

**Group**	**Total number (cases)**	**Number of pregnancies (cases)**	**Failure cases (cases)**	**Natural pregnancy success rate (%)**
Mild adhesion group	86	62	24	72.09
Moderate adhesion group	77	40	37	51.94
Severe adhesion group	43	12	31	27.91
Total	206	114	92	55.34

*Chi-square value 23.218*.

*P < 0.001*.

### Twisted Fallopian Tubes and Postoperative Pregnancy Outcomes

Of the 122 patients with no fallopian tube distortion, 65 had a spontaneous pregnancy within 2 years after the operation (natural pregnancy rate = 53.28%). A total of 156 patients had twisted/folded fallopian tubes, with 103 cases of partially folded fallopian tubes, 56 of which had a spontaneous pregnancy within 2 years after the operation (natural pregnancy rate = 54.37%). Another 53 patients had completely folded fallopian tubes, eight of which had a spontaneous pregnancy within 2 years after the operation (natural pregnancy rate = 15.09%). These results show that the impact on postoperative natural pregnancy is greater as the severity of fallopian tube distortion increases. The natural pregnancy rate was significantly lower in patients with completely folded fallopian tubes than in those with partially folded fallopian tubes and those with no distortion, and the difference was statistically significant (χ^2^ = 25.836, *P* < 0.001) (see Table 2).

**Table 2:** Fallopian tube distortion and post-operative pregnancy outcome.

**Group**	**Total number (cases)**	**Number of pregnancies (cases)**	**Failure cases (cases)**	**Natural pregnancy success rate (%)**
Non-folded fallopian tube group	122	65	57	53.28
Partially folded fallopian tube group	103	56	47	54.37
Completely folded fallopian tube group	53	8	45	15.09
Total	278	129	149	46.4

*Chi-square value = 25.836*.

*P < 0.001*.

### Hydrosalpinx and Postoperative Pregnancy Outcomes

Of the 99 patients with hydrosalpinx, 12 had mild hydrosalpinx, six of which had a spontaneous pregnancy within 2 years after the operation (natural pregnancy rate = 50%). Another 23 patients had moderate hydrosalpinx, four of which had a spontaneous pregnancy within 2 years after the operation (natural pregnancy rate = 17.39%), and 64 patients had severe hydrosalpinx, 10 of which had a spontaneous pregnancy within 2 years after the operation (natural pregnancy rate = 15.625%). These results show that the impact on postoperative natural pregnancy is greater as the severity of hydrosalpinx increases. The natural pregnancy rate was significantly lower in patients with severe hydrosalpinx than in those with mild or moderate hydrosalpinx, and the difference was statistically significant (χ^2^ = 7.554, *P* < 0.023) (see Table 3).

**Table 3:** Hydrosalpinx and postoperative pregnancy outcome.

**Group**	**Total number (cases)**	**Number of pregnancies (cases)**	**Failure cases (cases)**	**Natural pregnancy success rate (%)**
Mild hydrosalpinx group	12	6	6	50
Moderate hydrosalpinx group	23	4	19	17.39
Severe hydrosalpinx group	64	10	54	15.63
Total	99	20	79	20.2

*Chi-square value = 7.554*.

*P = 0.023*.

### Fallopian Tube Fimbriae Structure and Postoperative Pregnancy Outcomes

Of the 88 patients with intact fimbriae, 65 had a spontaneous pregnancy within 2 years after the operation (natural pregnancy rate = 73.86%). A total of 131 patients had retained fimbriae, 59 of which had a spontaneous pregnancy within 2 years after the operation (natural pregnancy rate = 45.045%). Another 59 patients had damaged fimbriae, five of which had a spontaneous pregnancy within 2 years after the operation (natural pregnancy rate = 8.47%). These results show that the impact on postoperative natural pregnancy is greater as the severity of fimbriae structure damage increases. The natural pregnancy rate was significantly lower in patients with damaged fimbriae than in those with retained and intact fimbriae, and the difference was statistically significant (χ^2^ = 60.907, *P* < 0.001) (see Table 4).

**Table 4:** Fallopian tube fimbria structure and post-operative pregnancy outcome.

**Group**	**Total number (cases)**	**Number of pregnancies (cases)**	**Failure cases (cases)**	**Natural pregnancy success rate (%)**
Intact fimbria structure group	88	65	23	73.86
Remained fimbria structure group	131	59	72	45.04
Damaged fimbria structure group	59	5	54	8.47
Total	278	129	149	46.4

*Chi-square value = 60.907*.

*P < 0.001*.

### The Correlation Between Fallopian Tube Lesions and the Incidence of Postoperative Ectopic Pregnancy

#### Pelvic Adhesions and Ectopic Pregnancy

In patients with pelvic adhesions, 114 got pregnant naturally after the operation. Of these 114, seven had an ectopic pregnancy (ectopic pregnancy rate = 6.14%). Of the seven patients with ectopic pregnancies, three had mild pelvic adhesions (ectopic pregnancy rate = 4.84%), two had moderate pelvic adhesions (ectopic pregnancy rate = 5%), and two had severe pelvic adhesions (ectopic pregnancy rate = 6.14%). There was no significant difference in ectopic pregnancy rates between the different degrees of pelvic adhesion (χ^2^ = 2.58, *P* = 0.275) (see Table 5).

**Table 5:** Pelvic adhesion and ectopic pregnancy.

**Group**	**Number of pregnancies (cases)**	**Ectopic pregnancy (cases)**	**Normal pregnancy (cases)**	**Ectopic pregnancy rate (%)**
Mild adhesion group	62	3	59	4.84
Moderate adhesion group	40	2	38	5
Severe adhesion group	12	2	10	16.67
Total	114	7	107	6.14

*Chi-square value = 2.58*.

*P = 0.275*.

#### Twisted/Wrapped Fallopian Tubes and Ectopic Pregnancy

In the group of patients with twisted/wrapped fallopian tubes, 129 got pregnant naturally after the operation. Of these 129, eight had an ectopic pregnancy (ectopic pregnancy rate = 6.2%). Of the eight patients with ectopic pregnancies, two had non-folded fallopian tubes (ectopic pregnancy rate = 3.08%), four had partially folded fallopian tubes (ectopic pregnancy rate = 7.14%), and two had completely folded fallopian tubes (ectopic pregnancy rate = 25%). These results indicate a correlation between fallopian tube distortion and ectopic pregnancy. The incidence of ectopic pregnancy increased with the severity of fallopian tube distortion, and the difference was statistically significant (χ^2^ = 6.036, *P* < 0.049) (see Table 6).

**Table 6:** Fallopian tube distortion and ectopic pregnancy.

**Group**	**Number of pregnancies (cases)**	**Ectopic pregnancy (cases)**	**Normal pregnancy (cases)**	**Ectopic pregnancy rate (%)**
Non-folded fallopian tube group	65	2	63	3.08
Partially folded fallopian tube group	56	4	52	7.14
Completely folded fallopian tube group	8	2	6	25
Total	129	8	121	6.20

*Chi-square value = 6.036*.

*P = 0.049*.

#### Hydrosalpinx and Ectopic Pregnancy

In patients with hydrosalpinx, 20 got pregnant naturally after the operation. Of the 20, eight had an ectopic pregnancy (ectopic pregnancy rate = 40%). Of the eight patients, two had mild hydrosalpinx (ectopic pregnancy rate = 33.33%), two had moderate hydrosalpinx (ectopic pregnancy rate = 50%), and four had severe hydrosalpinx (ectopic pregnancy rate = 40%). These results show that the rate of ectopic pregnancy was higher in patients with hydrosalpinx, but there was no significant difference between the different degrees of lesions (χ^2^ = 0.278, *P* = 0.87) (see Table 7).

**Table 7:** Hydrosalpinx and ectopic pregnancy.

**Group**	**Number of pregnancies (cases)**	**Ectopic pregnancy (cases)**	**Normal pregnancy (cases)**	**Ectopic pregnancy rate (%)**
Mild hydrosalpinx group	6	2	4	33.33
Moderate hydrosalpinx group	4	2	2	50
Severe hydrosalpinx group	10	4	6	40
Total	20	8	12	40

*Chi-square value = 0.278*.

*P = 0.87*.

#### Fallopian Tube Fimbriae Structure and Ectopic Pregnancy

In the fallopian tube fimbriae structure group, 129 patients got pregnant naturally after the operation. Of these 129, 30 had an ectopic pregnancy (ectopic pregnancy rate = 22.5%). Of the 30, four patients had intact fimbriae (ectopic pregnancy rate = 6.15%), 23 had retained fimbriae (ectopic pregnancy rate = 38.98%), and three had damaged fimbriae (ectopic pregnancy rate = 60%). These results indicate a correlation between the severity of fimbriae structure distortion and ectopic pregnancy. The incidence of ectopic pregnancy increased with the severity of fimbriae structure distortion, and the difference was statistically significant (χ^2^ = 22.611, *P* < 0.001) (see Table 8).

**Table 8:** Fallopian tube fimbria structure and ectopic pregnancy.

**Group**	**Number of pregnancies (cases)**	**Ectopic pregnancy (cases)**	**Normal pregnancy (cases)**	**Ectopic pregnancy rate (%)**
Intact fimbria structure group	65	4	61	6.15
Remained fimbria structure group	59	23	36	38.98
Damaged fimbria structure group	5	3	2	60
Total	129	30		23.26

*Chi-square value = 22.611*.

*P < 0.001*.

## Discussion

Pelvic adhesion is the leading cause of TFI. In the European Guidelines for the Prevention of Adhesion in Gynecological Surgery (2014), the European Society for Gynecological Endoscopy (ESGE) identified a clear correlation between adhesion and infertility (8). In the present study, the laparoscopic treatment of mild to moderate pelvic adhesions was effective, and it was found that the natural pregnancy rate increased as the severity of pelvic adhesion decreased. However, although laparoscopic pelvic adhesion lysis surgery is effective in treating mild to moderate pelvic adhesions, in patients with severe pelvic adhesions, it is not only difficult to restore the external anatomical structure of the fallopian tubes but also easy to cause new injury, thereby creating new adhesions. Moreover, the internal physiological function of the fallopian tubes has been seriously damaged in these patients, so the postoperative effect is not satisfactory. Therefore, for patients with severe pelvic adhesions, the purpose of surgery should be to create favorable conditions for subsequent treatment (such as assisted reproductive technology). After the operation, the time of a trial pregnancy and whether *in vitro* fertilization and embryo transfer (IVF-ET) could assist pregnancy should be determined based on the condition of the fallopian tubes and the age and economic status of the patient.

Fallopian tube distortion is another common cause of TFI. At present, there are no clear criteria for judging the degree of fallopian tube distortion. In this study, the patients were divided into groups based on the angle of fallopian tube distortion. The results showed that laparoscopic plastic repair of the fallopian tubes was effective in the treatment of partial distortion and can, therefore, significantly improve natural pregnancy rates. Increased fallopian tube distortion severity, however, caused a decrease in the natural pregnancy rate.

Hydrosalpinx is a pathological state of abnormal change in the shape of the fallopian tubes. It is caused by fimbriae adhesion, obstruction of the fallopian tubes, and the accumulation of oviduct fluid and inflammatory exudate in the lumen caused by chronic infection. Laparoscopic salpingostomy is an effective treatment for mild hydrosalpinx and can significantly improve the natural pregnancy rate. For patients with moderate or severe hydrosalpinx, however, it is difficult to achieve a satisfactory pregnancy rate even if the fallopian tubes are unobstructed after the operation due to irreversible damage to the function of the fallopian tubes. For these patients, fallopian tube ligation or resection is feasible, and assisted reproduction should be carried out. Patients with hydrosalpinx should be given individualized treatment based on a comprehensive evaluation and consideration of their age, number of years of infertility, infertility history, and type/severity of fallopian tube lesions.

The more serious the destruction of the fimbriae structure, the lower the postoperative pregnancy rate. Therefore, this study proposes that patients with more than two-thirds of residual fimbriae tissue after laparoscopic surgery can be recommended to continue with a trial pregnancy. However, if less than one-third of the residual fimbriae tissue remains, it is suggested that patients choose assisted reproduction. For younger patients, the duration of trial pregnancy can also be extended to 2 years after the operation. In addition, the number of residual fimbriae is closely related to pregnancy outcomes. Genital tract infections cause inflammation and adhesion in the fimbriae, and pelvic adhesions affect fallopian tube peristalsis, which can lead to ectopic pregnancy (9). Therefore, careful operation during laparoscopic surgery is vital to protect fimbriae tissue, thereby improving the natural pregnancy rate after the operation. If the fimbriae tissue is good, it should not be over-everted to avoid affecting its functional recovery and suturing and electrocoagulation should be avoided. Rational use of antibiotics to prevent and control postoperative infection, removing residual blood clots after the operation, and washing with a large amount of normal saline are conducive to reducing the incidence of adhesion and ectopic pregnancy (10). In addition, the use of medical anti-adhesion agents in the wound after laparoscopic surgery can avoid re-adhesion and the destruction of fimbriae tissue, effectively reducing the incidence of postoperative pelvic adhesion (11, 12).

The risk of postoperative ectopic pregnancy increases as the severity of fimbriae destruction and the degree of fallopian tube distortion increases. Therefore, reducing the damage to the fallopian tube mucosa during laparoscopic surgery, restoring the normal anatomical position of the fallopian tube as much as possible, actively applying anti-adhesion drugs, and encouraging the patient to get out of bed as soon as possible after the operation are conducive to improving the pregnancy outcome. Because it is very difficult to recover fallopian tube mucosa after pathological changes from surgical treatment, there is no ideal method for preventing postoperative ectopic pregnancy and re-adhesion. A solution to this problem requires further study.

This study provides guidance for the clinical treatment of patients with tubal infertility and analyzes the effectiveness of hysteroscopic and laparoscopic surgery in treating tubal infertility and the relationship between the degree of tubal lesions and the postoperative pregnancy rate. However, the current diagnosis and treatment methods are still difficult to relieve patients' complicated conditions. The establishment of a reliable clinical efficacy and prognosis evaluation system will bring benefits to all patients with tubal infertility. An excellent clinical efficacy and prognosis evaluation system is based on careful consideration, including fallopian tube morphology, pelvic adhesion, changes in the pelvic microenvironment, etc. This is also the limitation of this study: the lack of research on the pelvic microenvironment, including the pH of pelvic effusion and fallopian tube effusion, the concentration of inflammatory-related cytokines, sex hormone levels, and so on. Therefore, this study will improve the following directions in the future: 1. to further study the effect of pelvic microenvironment changes on postoperative pregnancy rate; 2. to establish a quantitative evaluation system of clinical efficacy and prognosis, and ultimately provide proper treatment for patients with tubal infertility.

## Data Availability Statement

The original contributions generated for the study are included in the article/supplementary material, further inquiries can be directed to the corresponding author/s.

## Ethics Statement

The studies involving human participants were reviewed and approved by Ethics Committee of The First People's Hospital of Yunnan Province. The patients/participants provided their written informed consent to participate in this study.

## Author Contributions

LN and D-HY conceived the idea and conceptualized the study. LN and JZ collected the data. HZ and C-FZ analyzed the data. M-FD and LN drafted the manuscript, then YA and D-HY reviewed the manuscript. All authors read and approved the final draft.

## Conflict of Interest

The authors declare that the research was conducted in the absence of any commercial or financial relationships that could be construed as a potential conflict of interest.

## Publisher's Note

All claims expressed in this article are solely those of the authors and do not necessarily represent those of their affiliated organizations, or those of the publisher, the editors and the reviewers. Any product that may be evaluated in this article, or claim that may be made by its manufacturer, is not guaranteed or endorsed by the publisher.
